# Developing a transparent reporting tool for AI-based diagnostic prediction models of disease and syndrome in Chinese medicine: a Delphi protocol

**DOI:** 10.3389/fdgth.2025.1575320

**Published:** 2025-05-16

**Authors:** Jieyun Li, Wei Song Seetoh, Jiekee Lim, Xin’ang Xiao, Kehu Yang, Si Yong Yeo, Boyun Sun, Jinhua Liu, Zhaoxia Xu, Linda L. D. Zhong

**Affiliations:** ^1^ School of Traditional Chinese Medicine, Shanghai University of Traditional Chinese Medicine, Shanghai, China; ^2^ Shanghai Key Laboratory of Health Identification and Assessment, Shanghai, China; ^3^ School of Biological Sciences, Nanyang Technological University, Singapore, Singapore; ^4^ Evidence Based Medicine Centre, School of Basic Medical Sciences, Lanzhou University, Lanzhou, China; ^5^ MedVisAl Lab, Lee Kong Chian School of Medicine, Nanyang Technological University, Singapore, Singapore; ^6^ Department of Gastroenterology, Longhua Hospital, Shanghai University of Traditional Chinese Medicine, Shanghai, China

**Keywords:** artificial intelligence, Chinese medicine diagnostics, reporting guideline development, Delphi method, predictive model

## Abstract

**Introduction:**

The application of artificial intelligence in diagnostic prediction models for diseases and syndromes in Chinese Medicine (CM) has been rapidly expanding, accompanied by a significant increase in related research publications. However, existing reporting guidelines for diagnostic prediction models are primarily tailored to Western medicine, which differs fundamentally from CM in its theoretical framework, terminology, and classification systems. To address this gap, it is essential to establish a transparent and standardized reporting tool specifically designed for CM diagnostic and syndrome prediction models. This will enhance the transparency, reproducibility, and clinical relevance of research findings in this emerging field.

**Methods:**

This study adopts a structured, multi-phase Delphi protocol. A core working group will first conduct a comprehensive review of published studies on CM diagnostic prediction models to develop an initial item pool for the Transparent Reporting Tool for AI-based Diagnostic Prediction Models of Disease and Syndrome in Chinese Medicine (TRAPODS-CM). Delphi questionnaires will then be distributed via email to a multidisciplinary panel of experts in CM, computer science, and evidence-based methodology who meet the inclusion criteria. The number of Delphi rounds will be determined by evaluating the active coefficient, expert authority, and expert consensus. Final consensus on the TRAPODS-CM checklist will be achieved through online meetings. The study will be governed by a Steering Committee, with the core working group responsible for implementation. After publication, the finalized checklist will be disseminated via multimedia platforms, seminars, and academic conferences to maximize its academic and clinical impact.

**Ethics and Dissemination:**

This project has received ethical approval from the National Natural Science Foundation of China (Grant No. 82374336) and the Institutional Review Board of Nanyang Technological University (IRB-2024-1007). The study findings will be disseminated through peer-reviewed publications.

## 1 Introduction

### 1.1 Background

Chinese Medicine (CM) diagnosis encompasses two core aspects: “Bing” (病, bìng, disease) and “Zheng” (证, zhèng, syndrome). While “Bing” corresponds to disease entities in Western medicine, “Zheng” emphasizes the imbalances in the body's functional state (1). For instance, a patient presenting with clear nasal discharge, fever, aversion to cold, and no thirst, accompanied by a pale red tongue and white coating, is diagnosed with wind-cold syndrome; whereas a patient exhibiting yellow nasal discharge, fever, thirst, a red tongue, yellow coating, and a rapid pulse is diagnosed with wind-heat syndrome. Although both conditions are classified as common cold in Western medicine, CM believes they represent different internal imbalances, that is, different syndrome types. While CM pays attention to pathological entities, it places more emphasis on a holistic perspective, comprehensively collecting the patient's clinical information through methods such as observation, auscultation and olfaction, inquiry, and palpation. These information are then analyzed through CM theories such as Yin-Yang and the circulation of Qi-Blood for comprehensive analysis to determine the specific syndrome type, thereby guiding the formulation of treatment plans and ultimately achieving the goal of restoring the body's internal balance (2). However, CM diagnosis often relies heavily on the practitioner's subjective experience, leading to inconsistencies and variability in diagnostic outcomes.

Artificial intelligence (AI) is emerging as a transformative player in healthcare, exhibiting a significant role in modernizing CM (3). To improve the accuracy, reliability, and scientific rigor of CM diagnosis, researchers are leveraging AI to develop objective diagnostic tools and employing machine learning techniques to build predictive models. For example, AI-based models have been used to assess lung cancer risk using tongue image features (4), classify CM syndromes in polycystic ovary syndrome (5), and predict blood pressure in deficiency syndromes using algorithmic approaches (6). These studies demonstrate the potential of AI in enhancing diagnostic standardization and treatment personalization. However, the reporting quality of existing studies on TCM syndrome-related prediction models remains sub-optimal. Preliminary research indicates that only 1.85% (7) of such studies mention relevant transparency reporting tools, including Transparent Reporting of a multivariable prediction model for Individual Prognosis Or Diagnosis (TRIPOD) (8) and Standards for Reporting of Diagnostic Accuracy Studies (STARD) (9). The TRIPOD + AI statement (10) extends TRIPOD by addressing machine learning-based western clinical prediction models. STARD focuses on evaluating sensitivity, specificity, and other metrics for Western clinical tools such as blood tests and imaging equipment. Neither of these tools adequately considers the holistic nature of disease and syndrome in CM or the theoretical framework underlying CM diagnostic reasoning. Therefore, the development of TRAPODS-CM aims to fill this gap by providing a tailored reporting standard for AI models in CM.

### 1.2 Rationale

The unique characteristics of CM syndromes, combined with the variability inherent in CM diagnosis, necessitate the development of a specialized transparent reporting tool for AI-based CM diagnostic prediction models. Existing tools such as TRIPOD and TRIPOD + AI, while valuable for Western medical applications, do not fully capture the theoretical and practical nuances of CM. Without tailored reporting guidelines, inconsistencies in study design, analysis, and interpretation may persist, limiting the reproducibility, validity, and clinical applicability of AI-based CM models.

Addressing these challenges require a systematic approach that integrates expertise from diverse fields, including CM clinicians, evidence-based medicine specialists, and computer scientists. By combining empirical knowledge with methodological rigor, it is possible to develop a reporting tool that meets the unique requirements of AI models in CM, ensuring their transparency, usability, and alignment with clinical practice.

### 1.3 Objectives

This study aims to develop the Transparent Reporting Tool for AI-Based Diagnostic Prediction Models of Disease and Syndrome in Chinese Medicine (TRAPODS-CM) through a Delphi method, engaging experts from CM, evidence-based medicine, and computer science to establish consensus on reporting standards tailored to CM diagnostic prediction models. The goal is to address the unique theoretical and practical aspects of CM while enhancing the transparency, reproducibility, and reliability of AI-based CM diagnostic models, ultimately promoting the integration of AI technology into CM clinical practice with standardized and actionable reporting guidelines that support both scientific rigor and clinical applicability.

## 2 Materials and methods

### 2.1 Study design

**Figure 1:**
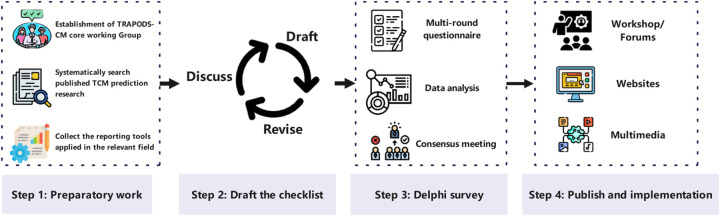
Process of TRAPODS-CM development. This figure was prepared using images from https://Flaticon.com.

The development of TRAPODS-CM follows the recommendations outlined in the CREDES (Recommendations for Conducting and Reporting of Delphi Studies) framework (11) and guidance from the EQUATOR Network (12). The Delphi method was chosen for its ability to gather diverse expert opinions, facilitate iterative feedback, and achieve consensus on complex topics. The study process comprises four main steps: preparatory work, drafting the checklist, conducting Delphi surveys, as well as publishing and implementing the final tool (Figure 1).

### 2.2 Steering committee and core working group

The project will be overseen by a Steering Committee consisting of senior researchers with expertise in Chinese Medicine diagnostics, evidence-based methodologies, and computer science. Their role includes supervising the research process and ensuring adherence to ethical and scientific standards but excludes participation as survey respondents.

The Core Working Group is responsible for executing tasks defined by the Steering Committee, including drafting the initial item pool, distributing questionnaires, analyzing data, and facilitating consensus meetings. This group will comprise five members: a CM clinician, a computer science specialist, an evidence-based medicine methodologist, and two doctoral candidates.

### 2.3 Questionnaire design and collection

The initial item pool for TRAPODS-CM was developed based on a prior systematic review (7) of published studies on diagnostic prediction models in CM. Reporting tools such as TRIPOD and STARD were identified and combined with CM-specific diagnostic content, resulting in 53 potential items across 10 domains (Table 1). Through multiple rounds of Delphi surveys and a final online meeting, experts will evaluate and confirm each item on the TRAPODS-CM checklist to ensure that it meets the basic requirements for high-quality, reproducible research reports on AI-related CM disease and syndrome prediction models.

**Table 1:** Initial item pool for TRAPODS-CM.

Domain	Item
Title	1 Title
Abstract	2a Brief Background
	2b Objectives
	2c Research design
	2d Data Sources
	2e Model development
	2f Model Validation
	2g Model Evaluation
	2h Data Description
	2i Model performance
	2j Key features
	2k Key findings
	2l Clinical significance
	2m Limitations
Introduction	3 Background
	4 Purpose
Methods	5a Research design
	5b Research design
	6a Data sources
	6b Data sources
	7 Predictive factors
	8a Outcome indicators
	8b Outcome indicators
	9 Sample size
	10 Missing data
	11a Data pre-processing
	11b Data pre-processing
	12a Feature selection
	12b Feature selection
	13 Machine learning algorithm
	14a Model training
	14b Model training
	15 Model evaluation
	16 Risk stratification
	17 Modelling verification
Results	18a Study subjects
	18b Study subjects
	18c Study subjects
	19 Model building
	20 Model structure
	21 Model efficiency
	22 Model optimization
Discuss	23 Explanation
	24 Limitations
	25 Significance
Conclusion	26 Conclusion
References	27 Reference
Other information	28 Funding support
	29 Acknowledgement
	30 Authors’ contribution statement
	31 Conflict of interest statement
	32 Data availability statement
Appendix	33 Appendix

**Figure 2:**
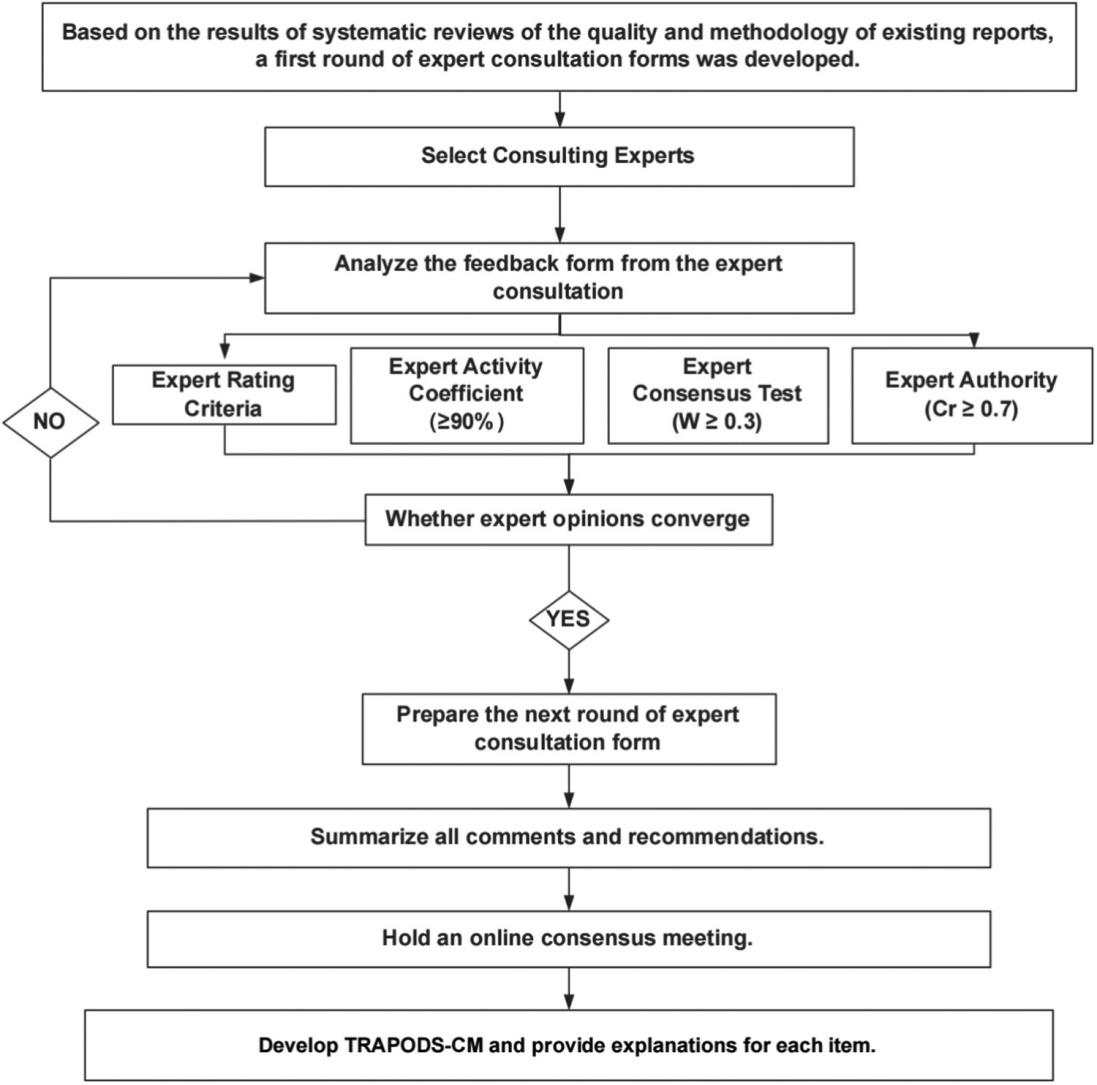
Flow chart of Delphi questionnaire design, distribution and expert consensus data collection.

As shown in Figure 2, the questionnaire distribution and data collection process are designed as follows:

(1) Expert Recruitment: Experts will be selected based on inclusion criteria, including domain-specific expertise, years of experience, and publication records. Invitations will be sent *via* email, accompanied by the ethically approved informed consent form (IRB-2024-1007). Confirmation of participation through email constitutes successful recruitment. All experts' personal information and response results will be kept safe and confidential.
(2) First-Round Delphi Questionnaire: Experts who confirm their participation will receive the first-round Delphi questionnaire, which available in both English and Chinese editions, consists of three parts:
  Section 1: Basic information about the expert.
  Section 2: The draft TRAPODS-CM checklist for evaluation.
  Section 3: Questions to assess familiarity with the content and judgment criteria.
(3) Subsequent Rounds: The core working group will compile all expert feedback collected in the previous round and, based on statistical analysis results, discuss it with the Steering Group to finalize the second-round item list. The finalized list will then be distributed via email to participating experts for further evaluation. We anticipate conducting 2–3 Delphi rounds, with the exact number determined by the convergence of expert opinions.
(4) Data Analysis and Iteration: Feedback from the first round will be analyzed using statistical methods. Two researchers will independently collate questionnaire data using Microsoft Excel 2019, and results will be cross-verified. Statistical analysis will be performed using SPSS 26 software. Revised items will be incorporated into subsequent rounds, and the number of rounds will depend on the convergence of expert opinions.

#### 2.3.1 Expert scoring criteria

Expert scoring is a method that combines qualitative and quantitative assessment (13). Based on the specific requirements of the evaluation object, several evaluation items are selected. Subsequently, evaluation criteria are developed for each item. Experts assign scores to each item based on their experience and the established criteria. Finally, the scores are statistically analyzed. This study uses a 5-point Likert scale (1–5), representing “Not at all important”, “Slightly important”, “Moderately important”, “Important”, and “Very important” respectively.

#### 2.3.2 Active coefficient

The active coefficient is generally represented by the questionnaire response rate (14). A higher response rate (≥90%) indicates greater expert engagement. Questionnaires with a high proportion of invalid responses or a high rate of missing data for key variables will be deemed invalid.

#### 2.3.3 Expert authority

Expert authority value (Cr) refers to an index used to measure the weight of an expert's opinion on the questions they answer in the decision-making or evaluation process (15). The Cr is jointly determined by the expert's judgment basis (Ca) and familiarity (Cs) with the problem. Specifically, experts need to rate their Ca and Cs based on the survey content. As shown in Table 2, experts should rate based on the importance of each indicator and quantify the ratings through the Likert 5-point scale method. Among them, Cs is divided into five levels: very familiar (1.0), relatively familiar (0.8), moderately familiar (0.6), not very familiar (0.4), and unfamiliar (0.2). Furthermore, as shown in Table 3, the Ca includes four dimensions: practical experience, theoretical analysis, understanding through domestic and foreign peers, and intuition. The influence degree of each judgment dimension is divided into three levels: large, medium, and small, and corresponding quantitative values are assigned. The calculation formula for the expert authority value is Cr = (Ca + Cs)/2. When Cr ≥ 0.7, the expert is considered to have a high authority, and their opinions will be highly valued in the subsequent analysis.

**Table 2:** Indicator importance rating levels.

Importance Rating	Score	Weighted value
Very important	5	1
Important	4	0.8
Moderately important	3	0.6
Slightly important	2	0.4
Not at all important	1	0.2

**Table 3:** Expert judgement basis (Ca) rating levels.

Expert judgement basis (Ca)	Influence degree		
	Large	Medium	Small
Practical experience	0.5	0.4	0.3
Theoretical analysis	0.3	0.2	0.1
Through domestic and foreign counterparts to understand	0.1	0.1	0.1
Intuition	0.1	0.1	0.1
Total	1.0	0.8	0.6

#### 2.3.4 Expert consensus test

The Kendall coefficient of concordance (W) is used to assess the level of agreement among experts. A *W* value between 0.300 and 0.500 with *P* < 0.05 indicates good consensus (16).

(4) Final Consensus Meeting: Once sufficient agreement is achieved, an online meeting will be held. During the meeting, each item in the checklist will be reviewed and finalized. This process will result in the creation of the final TRAPODS-CM checklist.

### 2.5 Inclusion and exclusion criteria of participants

#### 2.5.1 Sample size

There is no strict formula for determining the sample size in Delphi studies. A common range of 25–35 experts is often recommended, with adjustments based on the study's complexity and objectives (17). For this study, we aim to include at least 10 experts from each of the three fields: CM, evidence-based medicine, and computer science. In order to ensure that the consensus gathered would be generalizable, we will intentionally take the effort to send invitations to experts in different countries and regions so as to minimize the potential impact of restricted geographical representation within the expert panel. This distribution ensures a diverse and balanced pool of participants, while maintaining a manageable size for iterative rounds of feedback.

#### 2.5.2 Inclusion criteria

(1) CM Experts: Licensed CM practitioners with at least 5 years of clinical experience holding a senior professional title or affiliated with a renowned CM institution/university.
(2) Evidence-based Medicine Experts: Researchers with at least 5 years of experience in evidence-based medicine research and at least 5 scholarly publications in related fields.
(3) Computer Science Experts: Researchers with at least 5 years of experience specializing in machine learning, artificial intelligence, or related fields, and at least 3 publications related to machine learning.
(4) Willingness to Participate: Willing to participate in the entire Delphi study process, including multiple rounds of questionnaires and feedback.
(5) Language Proficiency: Able to communicate effectively in the designated language of the study (e.g., Chinese or English).

#### 2.5.3 Exclusion criteria

(1) Conflict of Interest: Having a conflict of interest with the research funder or related institutions.
(2) Inability to Participate: Unable to complete the entire Delphi study process due to health conditions, scheduling conflicts, or other reasons.
(3) Lack of Relevant Experience: Lacking sufficient experience or knowledge in the application of machine learning to TCM prognosis/diagnosis, despite being an expert in a related field.

## 3 Ethical considerations

This study has received ethical approval from the National Natural Science Foundation of China (Grant No. 82374336) and the Institutional Review Board of Nanyang Technological University (IRB-2024-1007). All participants will provide informed consent before participating in the Delphi rounds. The informed consent form will outline the study objectives, methods, and the participants' rights, including voluntary participation and the ability to withdraw at any stage without penalty. Anonymity and confidentiality will be ensured throughout the study by assigning unique codes to each participant and securely storing all data. The data will be retained in the private database of Shanghai University of Traditional Chinese Medicine. Additionally, to minimize group-think and social pressure during the Delphi process, expert feedback will remain anonymous across all rounds. These measures are intended to uphold ethical research standards and ensure the credibility and integrity of the study's outcomes. The development of the TRAPODS-CM reporting guideline focuses on expert consensus through the Delphi method, which primarily involves specialists in CM, evidence-based medicine, and computer science. Patients and the public were not directly engaged in this research's design, conduct, reporting, or dissemination plans.

## 4 Discussion

### 4.1 Implications

AI technology is gradually becoming an indispensable component of the modern CM diagnosis and treatment system. However, the CM diagnostic methods centered on “disease” and “syndrome” pose unique challenges for the application of AI due to their complexity (18, 19). This study aims to fill the critical gap in standardization and transparency in the field of CM diagnosis by developing a transparent reporting tool specifically for AI-based diagnostic prediction models. Based on our team's previous research (7), most related studies lack transparency, reproducibility, and clinical applicability, and fail to provide necessary clarity and systematic standards. Therefore, the design goal of TRAPODS-CM is to address this deficiency. Although existing guidelines such as STARD, TRIPOD, and TRIPOD + AI provide some guidance frameworks, they do not fully address the holistic and dynamic characteristics of TCM syndromes. In contrast, TRAPODS-CM, by focusing on the technical details of AI, can significantly enhance the transparency and reproducibility of TCM research and increase the trust of TCM clinicians in AI diagnostic tools. The development of TRAPODS-CM has the potential to revolutionize the integration of AI in CM diagnostics (20).

The core objective of TRAPODS-CM is to ensure that related research adheres to strict reporting standards, thereby meeting the fundamental requirements of high-quality and highly reproducible research. Its coverage encompasses the entire research process, from the design of CM studies, the collection of CM clinical data, to the data processing and validation based on AI. It is expected that this tool will provide CM researchers with a clear and standardized reporting framework, thereby enhancing the comparability and universality of research results. This not only facilitates rigorous assessment during the peer review process but also supports the development of clinically applicable models. Furthermore, by promoting transparency and adherence to ethical principles, TRAPODS-CM is expected to strengthen public and professional trust in the application of AI technologies in CM diagnostics, whilst also fostering their adoption in clinical practice of CM. Ultimately, these advancements is expected to contribute to improved diagnostic accuracy and efficiency in CM, thereby directly benefiting patients by providing more reliable and effective care.

### 4.2 Strengths and limitations

Protocols serve as a key step in enhancing the transparency and reproducibility of research, laying a solid foundation for the subsequent development and validation of tools. This study is currently at the protocol stage of the TRAPODS-CM tool development and has not yet completed the actual development and validation work of the tool, thus lacking specific implementation results. However, this study has several strengths, including its interdisciplinary approach, which integrates expertise from CM, evidence-based medicine, and computer science to ensure scientifically robust and clinically relevant guidelines. The methodology adheres to established standards like CREDES and EQUATOR, ensuring a systematic and rigorous process. Additionally, the tailored design of TRAPODS-CM addresses the unique characteristics of CM diagnostic prediction models, making it highly applicable to clinical realities. However, limitations exist, such as potential expert selection bias, where an unbalanced representation could limit the diversity of insights. There is also the risk of groupthink during Delphi iterations, which might suppress minority or innovative opinions despite anonymize responses. Furthermore, although efforts would be made to invite authoritative experts from different countries and regions to participate, due to the possible influence of regional factors on the discipline of traditional Chinese medicine, the expert panel might still consist of largely China-based experts. In the future validation stage, we will strive to attract a wider range of international participants to further enhance its global applicability and relevance.

## 5 Conclusion

The TRAPODS-CM reporting tool is currently in the development stages. This protocol provides a solid foundation for the subsequent development of the tool. Using a rigorous Delphi methodology, TRAPODS-CM will integrate expert consensus from diverse fields, ensuring it is both scientifically robust and clinically relevant. Each item on the checklist will be strictly evaluated and comprehensively discussed by the team of expert participants to reach a consensus, collectively forming the core elements of high-quality research reports, thereby supporting the scientific validation and clinical transformation of AI diagnostic models in CM. TRAPODS-CM is expected to provide a systematic framework for the evaluation, comparison, and optimization of CM AI diagnostic models, thereby promoting their wide application and efficient transformation in clinical practice. Future research will further validate and optimize this tool through extensive international cooperation to ensure its applicability and universality worldwide, thereby promoting the deep integration of CM and modern technology.
